# The Immuno-Regulatory Effects of *Schisandra chinensis* and Its Constituents on Human Monocytic Leukemia Cells

**DOI:** 10.3390/molecules16064836

**Published:** 2011-06-10

**Authors:** Rong-Dih Lin, Yi-Wen Mao, Sy-Jye Leu, Ching-Yi Huang, Mei-Hsien Lee

**Affiliations:** 1 Department of Internal Medicine, Ho-Ping Branch, Taipei City Hospital, Taipei 100, Taiwan; Email: lrd678@gmail.com (R.-D.L.); 2 Graduate Institute of Pharmacognosy, College of Pharmacy, Taipei Medical University, Taipei 110, Taiwan; Email: cutemaomao2007@gmail.com (Y.-W.M.); louies68@tmu.edu.tw (C.-Y.H.); 3 School of Pharmacy, College of Pharmacy, Taipei Medical University, Taipei 110, Taiwan; 4 Department of Microbiology and Immunology, Taipei Medical University, Taipei 110, Taiwan; Email: cmbsycl@tmu.edu.tw (S.-J.L.); 5 Center for Reproductive Medicine & Sciences, Taipei Medical University Hospital, Taipei 110, Taiwan

**Keywords:** schizandrin, gomisin A, macrophage inflammatory protein-1β, granulocyte-macrophage-colony stimulating factor, polymerase chain reaction

## Abstract

Many diseases occur when the immune system is weakened. Intracellular signals activate immuno-responsive cells to produce cytokines that modulate the immune response. *Schisandra chinensis* has been used traditionally to treat general fatigue, neurasthenia, and spontaneous sweating. In the present study, the effect of constituents of *S. chinensis* on cytokine release by human monocytic leukemia cells (THP-1) was tested using microparticle-based flow cytometric analysis. Two major lignans, schizandrin (Sch) and gomisin A (Gom A), were identified and shown to induce interleukin (IL)-8, macrophage inflammatory protein-1β (MIP-1β), and granulocyte-macrophage-colony stimulating factor (GM-CSF) release by THP-1 cells. By reverse transcription polymerase chain reaction (RT-PCR) or quantitative real-time PCR, there was a dose-dependent increase of *IL-8*, *MIP-1β* and *GM-CSF* mRNA levels. Thus, Sch and Gom A from *S. chinensis* enhance cytokine release by THP-1 cells and this effect occurs through mRNA upregulation. Upregulation of MIP-1β and GM-CSF in particular may have clinical applications. Therefore, *S. chinensis* may be therapeutically beneficial by promoting humoral and cell-mediated immune responses.

## 1. Introduction

The interaction of tumor cells and host immunity is increasingly well known to play a important role during the multiple stages of carcinogenesis and the strategy of endogenous immune systems to study effective anticancer therapeutics [1]. Immunotherapeutic approaches to the treatment of cancer have evolved during the last decades [2]. The immune response to foreign antigens results in a rapid expansion of effector lymphocytes that are triggered by activation of a variety of cell-surface receptors. Increased intracellular signaling activates immune responsive cells and enhances their production of cytokines, which, in turn, go on to modulate immune responses. Thus, the balance between pro-inflammatory cytokines and anti-inflammatory cytokines probably plays a critical role in the defense against diseases [3]. 

Several reports have also suggested that cytokines can be effectively used to treat cancer [4]. Cells of the monocyte/macrophage lineage play an important role in the immune response, these cells produce soluble factors such as cytokines and chemokines coordinate the immune response [5]. Some of the cytokines currently under investigation with potential benefits as cancer therapies include IL-2, IL-12, IFN-γ, GM-CSF and TNF-α [6]. Macrophage inflammatory protein (MIP)-1 chemokines were reported to play the protective roles in cancer immunotherapy [7]. Thus, detection of signaling intermediates or changes in cytokine levels are useful indicators to determine if a pharmacological agent has immuno-modulatory effects [8]. 

*Schisandra chinensis* Baill. (Schisandraceae) is a well-known Traditional Chinese Medicine (TCM). It has been used as an anti-tussive, a tonic, a sedative agent and, in traditional medicine, to improve the liver function of patients with viral hepatitis [9]. The dried fruits of *S. chinensis* have been used for several thousand years in China. In Japan, Schisandra fruit is a widely used component of Kampo medicines and, in the United States, it is a dietary supplement [10]. The main constituents of *S. chinensis* are schizandrin derivatives, and the active principles are lignans with a dibenzocyclooctadiene skeleton [11]. These lignans have been shown to stimulate liver regeneration [12], inhibit hepatocarcinogenesis [13], lower elevated dysfunction-associated liver transaminases in plasma [14] and suppress lipid peroxidation [15].

In the present study, we sought to evaluate the therapeutic of nutritional agents providing beneficial effects by modulating the immune system. Therefore, the aim of the present study was to investigate the effects of *S. chinensis* constituents on cytokine release by using microparticle-based flow cytometric analysis, reverse transcription polymerase chain reaction (RT-PCR) or quantitative real-time PCR on monocytic leukemia THP-1 cells.

## 2. Results and Discussion

In the past *S. chinensis* has been used as an alternative medicine for the treatment of various liver diseases [16,17] and more recently as a dietary supplement [18,19]. *S. chinensis* had reported antioxidative [20,21,22], antimicrobial [21,23,24], and inhibited retinol-induced irritation and pro-inflammatory cytokine secretion [25,26] in previous papers. It was also used for the production of soft drinks and health foods. There were approximately seven major important lignans, including schizandrin and gomisin A, described in *Schisandra* plants [27].

### 2.1. Analysis of Cytokine Release from Cells Treated with S. chinensis by Microparticle-Based Flow Cytometry

In the present study, the effect of LPS (10 ng/mL) and a 95% ethanolic extract of *S. chinensis* (M-4; 100 μg/mL) on the release of 17 immunoregulatory cytokines by THP-1 monocytes was evaluated by a fluid-phase immunoassay (BioRad Laboratories) run on a Bio-Plex Suspension Array System (Bio-Plex 100 System). These cytokines included IL-1β, IL-2, IL-4, IL-5, IL-6, IL-7, IL-8, IL-10, IL-12 (p70), IL-13, IL-17, G-CSF, GM-CSF, IFN-γ, MCP-1 (MCAF), MIP-1β, and TNF-α. Significantly increased release of IL-8, G-CSF, MCP-1 (MCAF), MIP-1β, and TNF-α but not IL-1β, IL-2, IL-4, IL-5, IL-7, IL-10, IL-12 (p70), IL-13, IL-17, GM-CSF, IFN-γ, and TNF-α was detected in the culture supernatants of cells incubated for 24 h (Table 1). Significantly increased release of IL-8, GM-CSF, and MIP-1β was detected in culture supernatants of cells incubated with *S. chinensis* extract (Table 1). 

**Table 1:** Microparticle-based flow cytometric assay (pg/mL) of *Schisandra chinensis* extract.

	Med ^a^	LPS ^a^	M-4 ^a^
IL-1β	ND	8.20 ± 1.77	ND
IL-2	0.52 ± 0.21	ND	ND
IL-4	ND	ND	ND
IL-5	ND	ND	ND
IL-6	ND	11.19 ± 5.13	ND
IL-7	ND	ND	ND
IL-8	3.36 ± 0.48	4515.95 ± 445.08 *	14.27 ± 5.93 *^,^^ #^
IL-10	ND	ND	ND
IL-12 (p70)	ND	ND	ND
IL-13	ND	0.68 ± 0.21	ND
IL-17	ND	4.32 ± 0.59	ND
G-CSF	2.02 ± 0.49	5.37 ± 1.66 *	ND
GM-CSF	ND	5.60 ± 1.45	27.91 ± 1.73 ^#^
IFN-γ	2.45 ± 0.83	31.13 ± 7.29 *	ND
MCP-1 (MCAF)	2.78 ± 0.89	2159.28 ± 245.11 *	7.30 ± 1.36
MIP-1β	6.62 ± 1.73	359094.48 ± 3286.85 *	22.27 ± 2.97 *^,^^ #^
TNF-α	ND	173.18 ± 34.83	ND

^a^ Abbreviations: Med, medium only; LPS, lipopolysaccharide (10 ng/mL); M-4, ethanolic extract of *S. chinensis* (100 μg/mL);*, ^#^ The data showed statistical significance (at *P*-value < 0.05) using the non-parametric Mann-Whitney *U*-test (* compared with Med, ^#^ compared with LPS). ND: It means that the data was out of the standard curve of the assay. The standard curves were ranging from 1.95 to 32,000 pg/mL by serial dilution of the reconstituted lyophilized standard.

In a previous paper, the production of inflammatory cytokines was investigated in the culture media of U937 and THP-1 cells with different flow cytometric bead-based assays [28]. Since the platform was well adapted to measure variations of cytokine secretion in the culture media, we first evaluated the *in vitro* proinflammatory properties of *S. chinensis* using microparticle-based flow cytometric analysis.

### 2.2. Analysis of the Isolated Compounds from S. chinensis

**Figure 1:**
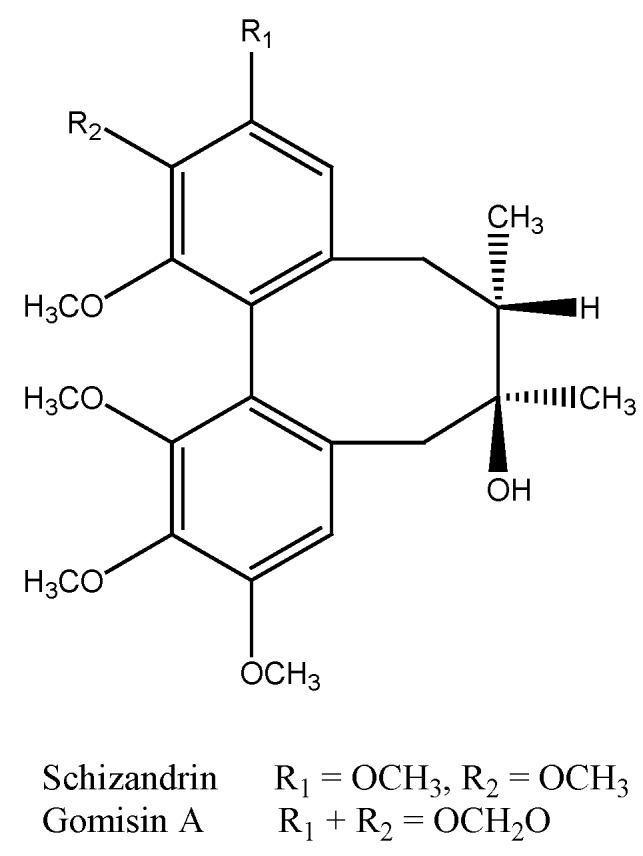
Structures of the active constituents isolated from *S. chinensis.*

**Figure 2:**
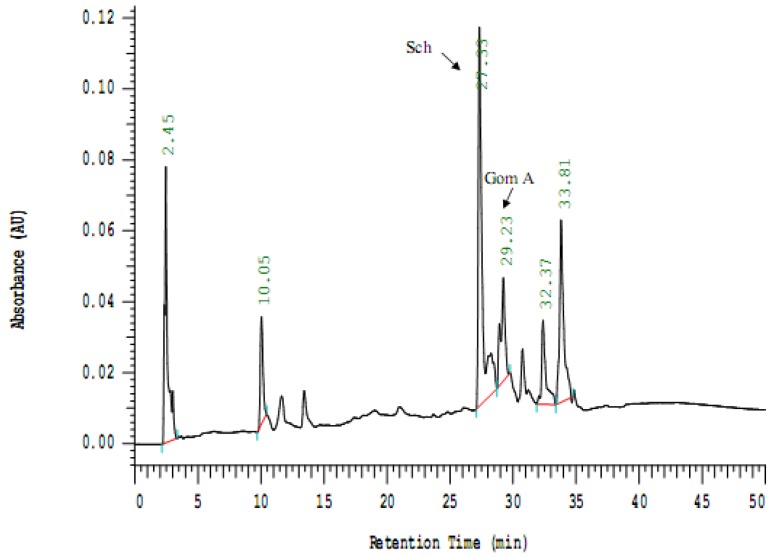
HPLC chromatogram of a 95% ethanol extract of *S. chinensis*. The analysis conditions were: column, Cosmosil 5C18-AR-II 4.6×250 mm (Nacalai Tesque); flow rate, 1 mL/min; detector, 254 nm; solvent system, 0–30 min, 5% MeOH to 100% MeOH; 30–50 min, 100% MeOH. Peak identifications: Sch, schizandrin; Gom A, gomisin A.

Two major constituents were isolated from *S. chinensis* in this study. The physical and spectral data of the two constituents were compared with previous literatures, and they were identified as gomisin A and schizandrin, respectively (Figure 1). The isolated compounds were also analyzed by HPLC (Figure 2). Retention times (in minutes) of the isolated compounds were as follows: schizandrin (Sch, 27.33) and gomisin A (Gom A, 29.23). The contents of schizandrin and gomisin A in *S. chinensis* extract were 36.8 mg/g and 28.1 mg/g, respectively.

### 2.3. Cell Viability of S. chinensis and Its Constituents in THP-1 Cells

MTT assay showed that the *S. chinensis* extract (100 μg/mL) had low cytotoxicity (cell viability, 102.01 ± 2.66%). The cell viabilities of THP-1 cells treated with the two isolated constituents, schizandrin and gomisin A, were also analyzed. When the concentration was 100 μM, both schizandrin and gomisin A showed low cytotoxicity (cell viability, 102.61 ± 4.96% and 93.18 ± 2.59%, respectively). After this low cytotoxicity was identified as a first test of possible clinical usefulness, the appropriate samples were further examined for their effects regulating cytokines in THP-1 cells. 

### 2.4. Analysis of Cytokine Release from Cells Treated with the Constituents of S. chinensis by Microparticle-Based Flow Cytometry

Since the release of only four cytokines (IL-8, GM-CSF, MCP-1 (MCAF), MIP-1β) was affected in our analysis system, only these four were assayed in the study of schizandrin and gomisin A. Flow cytometric assay found that both schizandrin and gomisin A significantly increased IL-8, GM-CSF, and MIP-1β release (Table 2). 

**Table 2:** Microparticle-based flow cytometric assay (pg/mL) of *Schisandra chinensis* components.

	Med	LPS	Sch^ a^	Gom A^ a^
		(10 ng/mL)	(100 μM)	(100 μM)
IL-8	6.843 ± 1.03	6493.99 ± 386.38 *	136.21 ± 24.10 *^, #^	67.15 ± 1.48 *^, #^
GM-CSF	ND	8.71 ± 3.77	17.45 ± 21.16 ^#^	12.37 ± 5.21 ^#^
MCP-1 (MCAF)	5.87 ± 1.44	2092.61 ± 208.26 *	9.20 ± 1.27	7.14 ± 2.84
MIP-1β	23.60 ± 3.67	19913.19 ± 3031.26 *	24.49 ± 5.97 *^, #^	21.30 ± 1.79 *^, #^

^a^ Abbreviations: Sch, schizandrin; Gom A, gomisin A. Sch: 100 μM (43.3 μg/mL); Gom A: 100 μM (41.6 μg/mL); *, ^#^ The data showed statistical significance (at *P*-value < 0.05) using the non-parametric Mann-Whitney *U*-test (* compared with Med, ^#^ compared with LPS). ND: It means that the data was out of the standard curve of the assay. The standard curves were ranging from 1.95 to 32,000 pg/mL by serial dilution of the reconstituted lyophilized standard.

### 2.5. Expression of IL-8, MIP-1β, and GM-CSF mRNA in S. chinensis and Its Constituents-Treated THP-1 Cells

**Figure 3:**
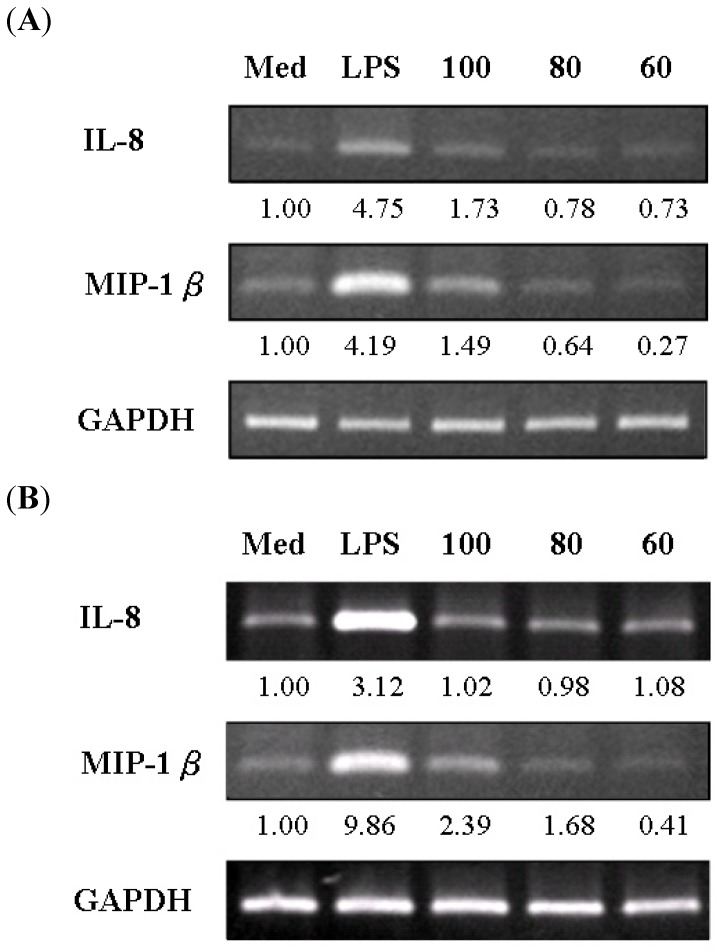
Expression of *MIP-1β* and *IL-8* mRNAs in THP-1 cells treated with various concentrations (μM) of (**A**) schizandrin (Sch) and (**B**) gomisin A (Gom A) as determined by RT-PCR analysis. The findings were normalized to the expression of *GAPDH* mRNA. Measurements were conducted in triplicate. Med: medium; LPS: 10 ng/mL.

**Figure 4:**
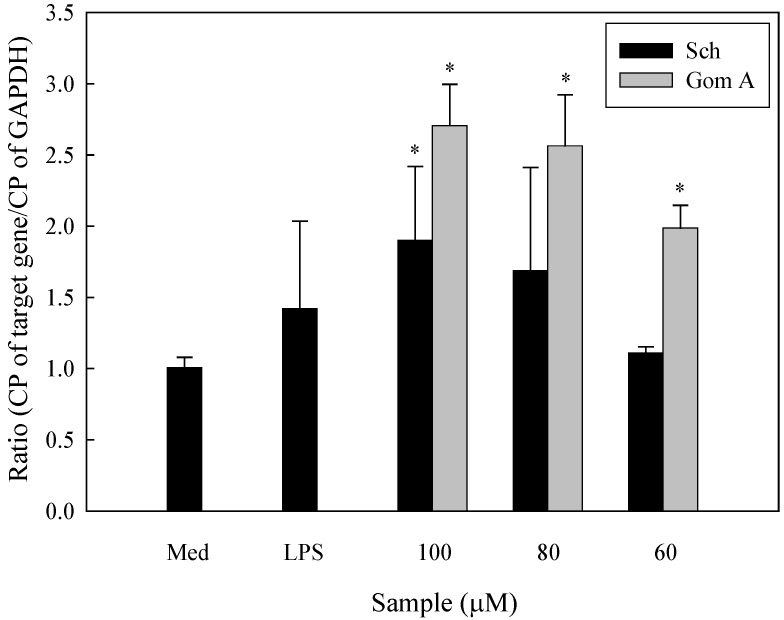
Expression of *GM-CSF* mRNAs in cells treated by schizandrin (Sch) and gomisin A (Gom A) using qRT PCR analysis (n = 3). Findings were normalized to the expression of *GAPDH* mRNA. Data were analyzed for statistical significance (*P* < 0.05, compared with medium only) using the nonparametric Mann-Whitney *U*-test. Med: medium only; LPS: 10 ng/mL.

To further determine whether the isolated constituents of *S. chinensis* induced cytokine expression at the mRNA level, total cellular RNA was extracted from THP-1 cells cultured in the presence or absence of the constituents and used as a template for RT-PCR. The *GAPDH* gene served as the housekeeping gene. The results showed that schizandrin upregulated both *IL-8* and *MIP-1β* mRNA expression and gomisin A upregulated *MIP-1β* mRNA expression at 100 μM. Laser densitometry analysis demonstrated that the *IL-8* mRNA to *GAPDH* mRNA ratio was 1.73-, 0.78-, and 0.73-fold for schizandrin (100, 80, and 60 μM, respectively) and 1.02-, 0.98-, and 1.08-fold for gomisin A (100, 80, and 60 μM, respectively), while the *MIP-1β* mRNA to *GAPDH* mRNA ratio was 1.49-, 0.64-, and 0.27-fold for schizandrin and 2.39-, 1.68-, and 0.41-fold for gomisin A (Figure 3A and Figure 3B). qRT-PCR found a 1.90-fold induction by schizandrin (100 μM), and dose-dependent, 2.71, 2.56, and 1.99 -fold induction by gomisin A (100, 80, and 60 μM, respectively) (Figure 4), relative to untreated control values. 

IL-8 may play an important role in chlamydial immunity or immunopathology [29]. Continuous IL-8 production can promote the infiltration of neutrophils. These neutrophils are not only inefficient in resolving chlamydial infections but also release proteases that damage cells. MIP-1β is a chemokine that can enhance the development of humoral, cellular mucosal, and systemic immunity. Its effects differentially regulate costimulatory molecule expression for support of humoral and cell-mediated immune responses [30]. GM-CSF regulates growth, differentiation, and activation of hematopoietic cells of multiple lineages [31]. It can attract and activate eosinophils, and stimulate the proliferation of and prolong the survival of hematopoietic cells. For patients immunosuppressed by HIV infection, malignancy, transplantation, or therapeutically, impaired host immunity against infections remains the main cause of morbidity and mortality. GM-CSF has been reported to increase the respiratory burst of human neutrophils *in vitro* following liver transplantation [32], restored the ability of monocytes from septic patients to respond [33], and compensated for the hypo-responsiveness of whole blood induced by trauma, sepsis, or cardiac surgery [34]. 

**Figure 5:**
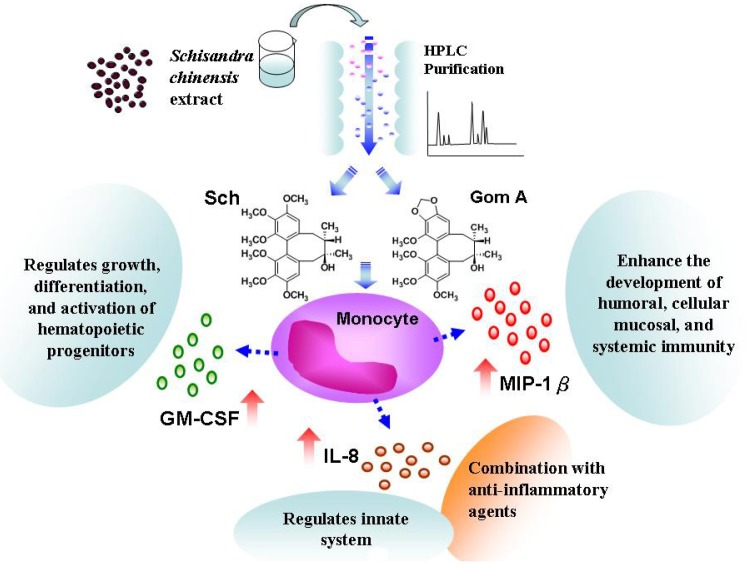
Putative immuno-regulatory effects of *S. chinensis* and its constituents on THP-1 cells.

Therefore, *S. chinensis* and its constituents may improve immunity by stimulating the release of these cytokines. Though the *S. chinensis* and its active constituents might increase IL-8 level in patients with HIV, however, the potential beneficial effect of GM-CSF to the patients were discussed in the paper. The combination therapy with anti-inflammatory agents or blocking IL-8 secretion in patients (e.g., patients with HIV) will be helpful in the clinical application. Therefore, *S. chinensis* might provide one of the additional supporting treatments for patients with immunosuppression (Figure 5).

## 3. Experimental

### 3.1. Materials

The dried mature fruits of *Schisandra chinensis* (Turcz.) Baill. (Schisandraceae) were purchased from a Chinese drug market in Taipei, Taiwan, and were identified by Dr. H.C. Chang, Bureau of Food and Drug Analysis, Department of Health, Taiwan. A reference specimen (Number M-4) was deposited in the Graduate Institute of Pharmacognosy, Taipei Medical University, Taiwan.

### 3.2. Chemicals and Reagents

3-(4,5-Dimethylthiazol-2-yl)-2,5-diphenyltetrazolium bromide (MTT), ethylenediaminetetraacetic acid (EDTA), agarose, L-glutamine, and 2-mercaptoethanol were purchased from Sigma-Aldrich (St. Louis, MO, USA). The Bio-Plex Human Cytokine 17-Plex panel was used with the Bio-Plex Suspension Array System (BioRad Laboratories Inc., Hercules, CA, USA). All chemicals and reagents used in the study were high-grade commercial products. Other materials included DPBS (Dulbecco’s phosphate buffered saline) (Sigma-Aldrich), TRIzol reagent, SuperScript TM II reverse transcriptase (Invitrogen, USA), High Pure RNA Isolation Kit (Roche), dNTP, DTT, DNA polymerase (Yeastern, Taipei, Taiwan), Oligo dT (Mission Biotech, Taipei, Taiwan), and LightCycler FastStart DNA Master SYBR Green I kit (Roche).

### 3.3. Isolation and Identification of Active Constituents of S. chinensis

The active constituents of *S. chinensis* were isolated according to a previously described procedure [35]. Briefly, the dried mature fruits of *S. chinensis* (600 g) were crushed and refluxed with ten-fold 95% ethanol for 8 h. The procedure was repeated twice. After filtration and combining the filtrates, the ethanol was evaporated. The aqueous concentrate was partitioned, in order, with *n*-hexane, ethyl acetate (EtOAc) and water-saturated *n*-butanol (*n*-BuOH). The *n*-hexane fraction was subjected to extensive silica gel column chromatography and eluted using an *n*-hexane:EtOAc gradient. Repeated chromatography of the fractions and monitoring by TLC and HPLC analysis provided the isolation of 2 constituents. Gomisin A and schizandrin were identified by comparisons with spectroscopic data in the literature [35].

### 3.4. HPLC Analysis

A Cosmosil 5C18-AR-II 4.6 × 250 mm column (Nacalai Tesque) was used, and the elution and isolation of compounds in the extract were monitored at 280 nm. Gradient elution was done with methanol (solvent A) and water (solvent B) at a constant flow rate of 1 mL/min. The profile was 5% A (0 min) to 100% A (30 min) and 100% A for 20 min. 

### 3.5. Preparation of Samples

All the test samples were concentrated under reduced pressure and freeze-dried. They were dissolved in DMSO (Sigma-Aldrich) and stored in a closed container until use. The final concentration of DMSO was less than 0.5% in medium. 

### 3.6. Cells and Cell Cultures

The human acute monocytic leukemia cell line THP-1 was obtained from the American Type Culture Collection (Rockville, MD). THP-1 cells were maintained in RPMI-1640 (Gibco, Grand Island, NY) supplemented with 10% fetal bovine serum (Sigma-Aldrich), L-glutamine (2 mmol/L), 2-mercaptoethanol (5 × 10^−5^ mol/L) and 1% penicillin/streptomycin (Sigma-Aldrich). The cells were grown at 37 °C in an environment of 5% CO_2_. 

### 3.7. MTT Assay

Cell viability was assessed by MTT assay [36]. THP-1 cells (1.0 × 10^5^ per-well) were suspended in RPMI 1640 medium with 10% FBS, dispensed in 24-well plates and incubated for 24 h. The cells were cultured for an additional 24 h with or without treatmesnts with the test samples. After centrifuging at 1,000 rpm for 10 min, the supernatants were removed. MTT (20 μL; 5 mg/mL; 3-(4,5-dimethylthiazol-2-yl)-2,5-diphenyltetrazolium bromide (MTT) from Sigma-Aldrich) and medium (180 μL) were added and cultured for 24 h, then transferred to Eppendorf tubes. After centrifuging at 1,000 rpm for 10 min and removing the supernatant, DMSO (200 μL) was added to the pellet. After thorough mixing, the mixture was transferred to an ELISA plate and results were read using a model µQuant microplate reader (Bio-Tek Instruments, Inc, Winooski, VT) with extinction measured at 570 nm. 

### 3.8. Cytokine Release from Cells by Microparticle-Based Flow Cytometric Analysis

We investigated the production of inflammatory cytokines in the culture media of THP-1 cells using different particle-based flow cytometric assays [37]. THP-1 cells were either untreated (negative control) or treated with 100 μg/mL of *S. chinensis* ethanolic extract or 100 μM of each *S. chinensis* isolated constituent for 24 h. As a positive control, cells were stimulated with 10 ng/mL of LPS (Sigma-Aldrich). The multiplexed analyses of cytokines with the Bio-Plex system used a liquid suspension array of 17 sets of 5.5-μm beads (Bio-Plex Human Cytokine 17-plex panel; Bio-Rad, Hercules, CA) internally dyed with different ratios of two spectrally distinct fluorochromes. Eight point standard curves (0.2 to 3,200 pg/mL) were obtained by serial dilutions of reconstituted lyophilized standards. Data were analyzed by Bio-Plex ManagerTM Software (Bio-Rad). Supernatants were frozen at –80 °C until analyzed simultaneously for the following 17 cytokines: IL-1β, IL-2, IL-4, IL-5, IL-6, IL-7, IL-8, IL-10, IL-12 (p70), IL-13, IL-17, G-CSF, GM-CSF, IFN-γ, MCP-1 (MCAF), MIP-1β, and TNF-α. These were also analyzed by 17-plex fluid-phase immunoassay using custom kits (BioRad Laboratories) run on a Bio-Plex Suspension Array System (Bio-Plex 100 System). Microsphere fluorescence was measured using a Luminex-100 cytometer (Luminex Corporation, Austin, TX, USA) equipped with StarStation software (Applied Cytometry Systems, Dinnington, UK) [38]. All samples were analyzed in triplicate. Cytokine levels were determined from a nonlinear regression routine, four-parameter logistic (4PL), and expressed as pg/mL. 

### 3.9. RNA Isolation and Reverse Transcription

Total RNA was isolated using a High Pure RNA Isolation Kit (Roche Molecular Systems Inc., Branchburg, NJ, USA) according to the manufacturer’s instructions. Total RNA purity was evaluated using the A260/A280 ratio. To prepare a cDNA pool from each RNA sample, total RNA (1 µg) was reverse transcribed using a Transcriptor First Strand cDNA Synthesis Kit (Roche). Each cDNA pool was stored at –20 °C until quantitative real-time polymerase chain reaction (qRT PCR) analysis was performed.

### 3.10. PCR Primers

The sequences of the oligonucleotides used in PCR reactions were as follows: IL-8, 5’-ATGACTTCCAAGCTGGCCGTG-3’ (forward), and 5’-TTATTGAATTCTCAGCCCTCTTCAAA AACTTCTC-3’ (reverse); MIP-1β, 5’-CCAAACCAACC GAAGCAAG C-3’ (forward) and 5’-ACAGTGGACCATCCCCATAG-3’ (reverse); GAPDH, 5’-ACCACAGTCCATGCCATCAC-3’ (forward) and 5’-TCCACCACCCTGTTGCTGTA-3’ (reverse).

### 3.11. Reverse Transcription-Polymerase Chain Reaction (RT-PCR)

THP-1 cells were treated with *S. chinensis* constituents for 6 h. Total RNA was extracted with TRIzol reagent (Invitrogen, Carlsbad, CA) and was reverse transcribed with SuperscriptⅡ (Invitrogen) according to the manufacturer's instructions. The resulting products were analyzed by electrophoresis on 1.5% agarose gels and stained with ethidium bromide. Specific primers for GAPDH were used as controls [39].

### 3.12. Quantitative Real-Time PCR (qRT-PCR)

Quantitative real-time PCR reactions were performed on a Roche LightCycler Instrument 2.0 using LightCycler DNA Master SYBR Green I kit (Roche). RNA was extracted using TRIzol reagent (Invitrogen Life Technologies). RNA (5 μg) was reverse transcribed using Superscript II reverse transcriptase (Invitrogen Life Technologies), and PCR amplification used a LightCycler DNA Master SYBR Green I kit (Roche). The PCR primer sequences used were as follows: GAPDH, 5’-ACACCC ACTCCTCCACCTTTG-3’ (forward) and 5’- GCTGTAGCCAAATTC GTTGTCATAC-3’ (reverse); GM-CSF, 5’-GAGTGAG ACCGGCCAGATGA-3’ (forward) and 5’-ACCC CTTGGTCCCTCC AA-3’ (reverse). The mRNA was normalized to GAPDH mRNA levels, and results are given as the fold-induction of mRNA expression relative to control samples. The parameters for PCR included denaturation at 95 °C for 10 min, followed by 45 cycles at 95 °C for 10 s, 60 °C for 5 s, 72 °C for 4 s, and was followed by a melting curve analysis beginning at 95 °C, continuing at 65 °C for 30 s, and finally returning to 95 °C. At the end of each RT-PCR run, the data were automatically analyzed and an amplification plot was generated for each cDNA sample. From these plots, the LightCycler4 Data analysis software automatically calculated the CP value (crossing point: the turning point corresponds to the first maximum of the second derivative curve) which indicates the beginning of exponential amplification. The mRNA level was normalized with reference to the amount of housekeeping gene transcript (GAPDH mRNA). 

### 3.13. Statistical Analysis

Results are expressed as Median (IQR, the interquartile range). Differences between the data sets were tested for significance by means of the non-parametric Mann-Whitney *U*-test. P < 0.05 was considered to indicate significantly different data sets.

## 4. Conclusions

*S. chinensis* and its constituents exhibit various immunoenhancing activities. In the present study, microparticle-based flow cytometric assay demonstrated that *S. chinensis* extract could upregulate cytokine expression in human acute monocytic leukemia cell line (THP-1). In particular, two isolated active constituents, schizandrin (Sch) and gomisin A (Gom A), caused increased MIP 1β and GM-CSF release and upregulated the level of *IL-8*, *MIP-1β* and *GM-CSF* mRNA transcription. *S. chinensis* may regulate growth, differentiation, and activation of hematopoietic progenitors as well as enhance the development of humoral, cellular mucosal, and systemic immunity (Figure 5). This is the first report to evaluate and individual constituents from *S. chinensis* that regulate cytokines in THP-1. The mechanism of schizandrin and gomisin A activity remains to be elucidated. *S. chinensis* and its constituents might be regarded as potential immuno-regulatory foods. 
